# Second-order instantaneous causal analysis of spontaneous MEG

**DOI:** 10.1162/imag_a_00553

**Published:** 2025-04-25

**Authors:** Yongjie Zhu, Lauri Parkkonen, Aapo Hyvärinen

**Affiliations:** Department of Computer Science, University of Helsinki, Helsinki, Finland; Department of Neuroscience and Biomedical Engineering, Aalto University School of Science, Espoo, Finland

**Keywords:** causality, autocorrelation, blind source separation, magnetoencephalography (MEG), effective connectivity, causal connectomes

## Abstract

Despite decades of research, discovering instantaneous causal relationships from observational brain imaging data, such as spontaneous MEG energies or fMRI, remains a difficult problem. Popular methods, such as Granger Causality and Non-Gaussian Structural Equation Models (SEM), either are unable to handle instantaneous effects or do not work because the data are not non-Gaussian enough. Here, we propose a model with instantaneous causality for temporally dependent variables; these are both very common properties in neuroimaging data. Then, we propose a method to estimate the causal directions based on likelihood ratios, which are related to mutual information between the residual and data variables. We thus construct a simple decision criterion that allows for instantaneous causal discovery in time-dependent data. The proposed method is computationally and conceptually very simple, and we show with simulated data that it performs well even in the case of limited sample sizes, presumably due to the general optimality properties of likelihood. We further apply it to an MEG dataset from the Cam-CAN repository, for which the method gives consistent causal directionalities of energies both intra-subject and inter-subject, as measured by split-half tests. It also gives better performance than Granger causality and non-Gaussian SEM methods in a brain age prediction task. The results also demonstrate that our method might be useful in analyzing causal brain connectomes in functional brain-imaging data.

## 1 Introduction

In network neuroscience, the prevailing approach within neuroimaging community is to characterize the connections between brain regions with statistical correlations (Biswal et al., 1995;Smith et al., 2011). The connections learned through these methods are referred to as “functional connectivity”. This term serves to distinguish them from “effective (or causal, directed) connectivity,” wherein one region or node is characterized as having a causal impact on another (Friston, 2009;Reid et al., 2019). Recently, a small but increasing community of neuroimaging researchers have been shifting emphasis from functional connectivity among neural time series toward effective connectivity estimated by causal discovery analysis (Rawls, et al., 2022,^2023^;Reid et al., 2019;Sanchez-Romero & Cole, 2021;Zhang & Hyvärinen, 2010).

A common method for causal discovery using neuroimaging data is Granger causality (GC), which has a long history in imaging neuroscience (Barnett & Seth, 2014;Seth et al., 2015). However, GC has well-known limitations, such as a lack of ability to account for instantaneous (or contemporaneous) causal relationships between variables (Arab et al., 2023). This is especially important for slowly changing neuroimaging signals, such as fMRI and energies or envelopes of E/MEG (Arab et al., 2023;Hyvärinen & Smith, 2013;Hyvärinen et al., 2010;Ramkumar et al., 2014). Their time resolution is usually within a few hundred milliseconds to several seconds. This duration is roughly one order of magnitude slower compared to the time required for the underlying neural process to propagate across the brain (Nunez & Srinivasan, 2006;Sutton et al., 1965). Thus, there is a sufficient time frame for causal influences to propagate among the brain regions or nodes within the network, implicating that such causal interactions appear to be instantaneous, which makes the causal discovery significantly more challenging (Arab et al., 2023;Hyvärinen et al., 2010).

Structural equation models (SEM), which do not rely on time lags, are a popular alternative method to estimate the causal direction. Since it is known that SEM cannot be estimated for Gaussian data without strong prior knowledge, the crucial question is what additional information we need to estimate the model. Traditional approaches to SEM assume that only a few connections are possible based on a priori knowledge, and the directionality is typically fixed as well. This is, of course, unacceptable if our goal is to discover causal relationships in a data-driven way. However, it is possible to estimate SEM with only general assumptions using the non-Gaussianity of data, resulting in the linear non-Gaussian acyclic model (LiNGAM) (Shimizu et al., 2006). LiNGAM was shown to perform best in detailed simulations comparing many methods for SEM estimation (Hyvärinen & Smith, 2013;Shimizu et al., 2006). Yet, its performance was not very satisfactory on a single-subject level, unless the measurements were unrealistically long, or the non-Gaussianity of data was enough.

In fact, non-Gaussianity for the LiNGAM with no temporal structure is only one possible source of additional information that we could use to estimate multivariate models. For autocorrelated variables, another source of information might be the temporal dependencies (e.g., autocorrelations). The fact is that it is common for time series to be temporally autocorrelated, especially for brain imaging data, such as MEG and EEG. The temporal second-order statistics could be used for estimating SEM in such case. Meanwhile, in the theory of blind source separation, similar theories have appeared in the contexts of separation by autocorrelations based on stationary second-order statistics (Belouchrani et al., 1997). It is widely recognized in the theory of ICA that estimation of covariances (second-order statistics) is more efficient and less sensitive to outliers than estimation of measures of non-Gaussianity (Hyvärinen et al., 2001). The basic idea is to assume that the causal time series and the residuals in the model have distinct autocorrelation spectra, similar to using second-order blind source separation to estimate the model, which makes the causal direction identifiable.

In this paper, we propose a model with instantaneous causality and time-dependent variables, and then develop a quite simple method to estimate such causal models based on a pairwise approach inHyvärinen and Smith (2013). The approach uses the likelihood ratio, connected to mutual information between residual and data variables, of the models corresponding to the two directions of causal influence. We validate the method on simulation data and further apply it to a real MEG dataset to demonstrate the effectiveness of instantaneous causal discovery in parallel time series for analyzing energies of resting-state MEG data. The main contribution of this work lies in applying the proposed method to instantaneous causal analysis for the energies of MEG data. Specifically, we first introduced the causal model and estimation method tailored for some statistical properties prominent in MEG. Second, we applied the method to identify the directional connectivity of resting-state MEG and used the derived connectivity measures for brain-age prediction.

## 2 Methods

In this section, we present the new causal model for instantaneous causal discovery with time-dependent variables and develop its estimation. This section is structured as follows: We first define the problem inSection 2.1. We derive the estimation based on the ratio of likelihoods of the models with mutual information inSection 2.2. We show how to estimate the whole network with the two-stage method inSection 2.3.

### 2.1 Model definition

Consider the case of a bivariate time series, with variables$x_{1},\ldots \;,\;x_{n}$and$y_{1},\ldots \;,y_{n}$, both of lengthntime points. Denote the whole time series as the n-dimensional vectorsxandy. Assume further that we observe many instances of those time series. Thus, we have many observations of bothxandy. In case we actually have just two very long (potentially infinite) stationary time series, we can consider windows of those bivariate time series, and eachxandyis one window of n time points. Assume that for any fixedt, both$x_{t}$and$y_{t}$have been standardized.

Now, consider two causal models, between which we need to choose the true one based on observed data. The first expresses thatxcausesy, instantaneously as

$$\;y_{t}=\alpha x_{t}+d_{t},\;\text{or}\;\text{y}=\alpha \text{x}+\text{d}$$ (1)

with a regression coefficientα, and a random noise variable$d_{t}$. The second model expresses the causality in the opposite direction

$$x_{t}=\alpha y_{t}+e_{t},\;\text{or}\;\text{x}=\alpha \text{y}+\text{e}$$ (2)

It is well known (Hyvärinen & Smith, 2013) that the regression coefficient has to be equal in the two directions. In this paper, we assume the variables are all jointly Gaussian. Crucially, we further assume that the regressor in the true model has*autocorrelations*. This is what makes the causal direction identifiable. More precisely, the causal direction is identifiable if and only if the noise variable does not have the same autocorrelations as the true regressor: the autocorrelations need to be different for at least one time lag.

The identifiability essentially follows from the theory of second-order blind source separation (Belouchrani et al., 1997). Indeed, one way of approaching this problem would be to follow the approach of the original LiNGAM (Shimizu et al., 2006), which is to first estimate a linear latent variable model akin to ICA, and then infer the causal direction from the coefficients of the estimated de-mixing matrix. We could here use second-order blind source separation methods to estimate the model, instead of non-Gaussianity-based ICA as in LiNGAM. However, more direct approaches are possible due to the special properties of the causal models (Shimizu et al., 2011). In particular, here we followHyvärinen and Smith (2013)who showed how in the bivariate case, the problem can be solved by computing the ratio of likelihoods of the models corresponding to two directions of causality and connecting it to some statistics of the residuals and the data variables. Their approach was based on LiNGAM and thus used non-Gaussianity. Next, we show how to develop a similar theory for the case of Gaussian, autocorrelated variables. We must stress that assuming Gaussianity is only a working assumption, that is, it is a mathematical approach for building a method that uses only second-order statistics (covariances) and leads to a simple and efficient algorithm. In other words, the consequence of using a Gaussian model is that the resulting techniques are based on second-order statistics only. The algorithm obtained via this simplifying assumption is, in fact, applicable to non-Gaussian variables.

### 2.2 Estimation

Consider the mutual informationIbetweenxand the residuald. The mutual information is a principled measure of independence, and is defined in terms of the differential entropieshas

I(x, d)=h(x)+h(d)−h([x;d]) (3)

where[x;d]is the concatenation of the two vectors to form a 2*d*-dimensional random vector. Since both vectors are Gaussian, we can use the well-known formula of Gaussian entropy:

$$h(\text{x})=\frac{1}{2}\text{det}\;\text{cov}(x)+\frac{1}{2}\left(n+\text{log}{\left(2\pi \right)}^{n}\right)$$ (4)

which gives the mutual information as

2I(x, d)=logdetcov(x)+logdetcov(d)−logdetcov([x;d]) (5)

This is a rather simple function of the covariances ofx,d, and their concatenated matrix[x;d]. The corresponding formula can be readily developed forI(y, e).

A well-known idea in causal discovery is that in the true model, the residual is independent of the regressor, while in the wrong model, that does not hold. Thus, we can compare the mutual information in the two models to see which model exhibits such independence to a larger degree. To this end, we simply take the difference of the mutual information to arrive at the decision criterion:

C=2I(x, d)−2 I(y, e)=logdetcov(x)+logdetcov(d)−logdetcov([x;d]) −[logdetcov(y)+logdetcov(e)−logdetcov([y;e])]  (6)

which is remarkably simple to estimate, and the estimator is fast to compute. We propose the decision rule that if this quantity*C*is negative, we choose model (1), and if it is positive, we choose model (2).Hyvärinen and Smith (2013)further showed that such a difference in mutual information is equivalent to the log-likelihood ratio. Such a likelihood ratio is likely to provide a statistically powerful approach due to the general optimality properties of likelihood. In particular, the likelihood ratio test has maximum power over all tests with the same significance level, as stated by the classical Neyman-Pearson Lemma.

Since the method exploits only autocorrelations of variables and not non-Gaussianity, it could be particularly useful for time-dependent brain-imaging data. Neuroimaging data from fMRI, or energies of E/MEG data, also follow a practically instantaneous causality model as above, due to the slow temporal scale of the measurement system.

### 2.3 Effective connectivity networks with more than two variables

A well-known and popular method in neuroimaging analysis is to regard statistically correlations as connections between brain areas (Smith, 2012). The whole brain connections estimated by such methods are called functional connectivity networks. In contrast, effective connectivity networks can be seen as describing the causal directions of such functional connections. To estimate an effective connectivity network of*n*variables (or nodes/sources), we thus apply a two-stage approach that is well known in the literature (Hyvärinen & Smith, 2013;Hyvärinen et al., 2010). First, we estimate which nodes are connected using a covariance-based method, such as the L1-penalized inverse covariance (precision matrix). Here, we adopted graphical lasso estimator to estimate the sparse inverse covariance with cross-validation to automatically set the parameter of L1 penalty, which was implemented in the*Scikit-learn*python package with*sklearn.covariance.GraphicalLassoCV*function (Pedregosa et al., 2011). Next, for each pair of connected nodes, we estimate the direction using the pairwise approach proposed above. This combination of the information on the existence of connections given by the inverse covariance with the directionality given by the proposed pairwise method gives a method for estimating the whole effective connectivity networks.

## 3 Experiments

### 3.1 Simulations

#### 3.1.1 Data generation

We first applied the methods to artificial data generated according to the generative causal model. We modeled various effects, including source autocorrelations and measurement noise. The autoregressive (AR) model is used and offers a straightforward, flexible framework that allows us to simulate temporal dependencies in a controlled and interpretable way, making it well-suited to this specific methodological validation. First, a temporally dependent regressor signal (causal variable) was randomly generated according to a Gaussian AR process with first order (i.e., AR(1) model) using Gaussian innovations (we also used a higher order AR(3) to generate the data and run the analysis; the results were shown inSupplementary Material Fig. S5). The effect signal was generated with a regression coefficientαfrom the regressor in the causal model, and the random noise was also generated according to a Gaussian AR(1) process with taking an autoregressive coefficient of 0.2. Second, to examine the robustness of the methods to observational noises and innovations in the AR process, we add Gaussian observational noises to all the variables after generating the data using the AR model with Gaussian innovations and Laplace innovations, separately. The Laplace innovation would, to a certain extent, lead to the variables with non-Gaussian properties. We generated 10,000 data sets of data from the model with x → y with different sample sizes, and the performances were evaluated by computing the percentage of correct estimates for causal direction. It should be noted that we did not do simulation analysis on the networks estimated using the two-stage approach mentioned inSection 2.3, since the performance, in this case, depends too much on the method for undirected connectivity estimation, which has been analyzed in detail in existing literature (Brier et al., 2015;Mahadevan et al., 2021).

For comparison, we also applied a pairwise LiNGAM (pwLiNGAM) method based on the non-Gaussianity estimation (Hyvärinen & Smith, 2013), and a standard Granger Causality (GC) method with Akaike information criterion (aic) to determine the model order. Further, we investigated the robustness of the different autoregressive coefficients in the regressor (the strength of the temporal dependence), the regressor coefficients in the true model, non-Gaussian innovations, and the observational noises.

#### 3.1.2 Results

**Fig. 1.**
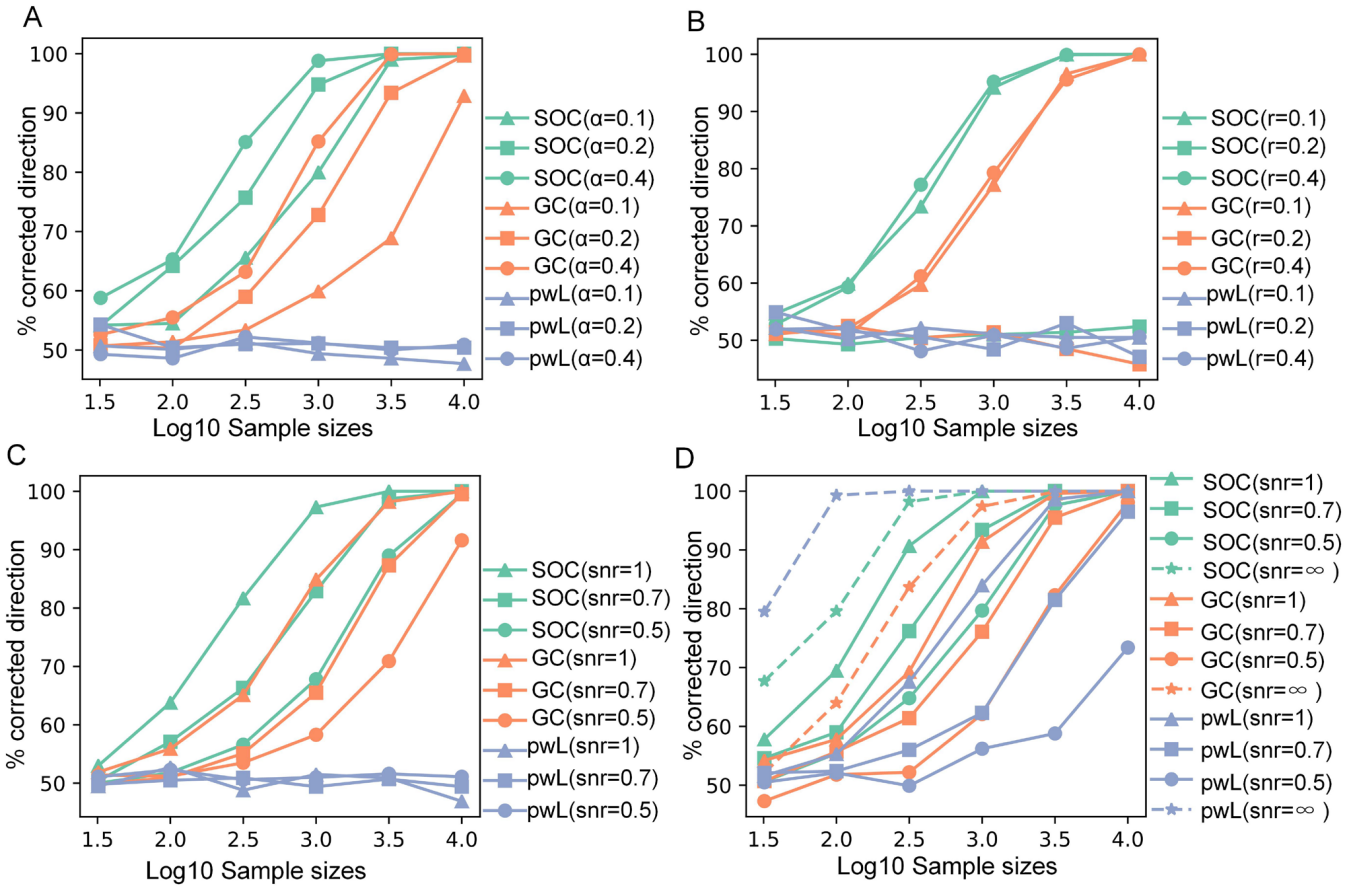
Results from simulated data (SOC: Second-Order Causality; GC: Granger Causality; pwL: pairwise LiNGAM). Percentage of directions found correctly among all connections estimated to exist. (A) the impact of the regression coefficients in the true model; (B) the impact of the autocorrelation in the regressor variables; (C) Gaussian innovations in AR model and with Gaussian observational noises. (D) Laplace innovations in AR model and with Gaussian observational noises.

The results are shown inFigure 1.Figure 1Ashows that the proposed method, second-order causal discovery (SOC), achieved higher direction decision accuracies that are consistently and significantly above chance level, indicating the model’s ability to reliably capture the causal directions within the data. This suggests that our estimation method effectively identifies directional information flows beyond random prediction, demonstrating promising potential for analysis of real data. We can see that the larger the regression coefficientαin the true model (high signal-to-noise ratio in the true model), the easier it makes the correct decision. The decision accuracy also goes down when the number of samples decreases. The pwLiNGAM method failed to correctly discriminate the causal direction since its estimation was based on the non-Gaussianity of the variables. Although the GC method performed well when the sample size was large enough, it failed when the sample size was quite small. However, the SOC achieved better performance when the number of time points is typically quite small, presumably because of the general optimality properties of likelihood.

Figure 1Bshows the impact of the autoregressive coefficientsrin the regressor variable on the performance. We can see that the performance was similar when the values autoregressive coefficientrvaried. However, the method failed withr=0.2since the variable had the same autocorrelations as the noise variable in the true causal model, which confirmed that the causal direction is identifiable if and only if the noise variable does not have the same autocorrelations. All the second-order methods would fail under the model when the variable and noise had the same autocorrelation spectra.

Figure 1C-Dshows the results of adding observational noises and having non-Gaussian innovations.Figure 1Cindicates that the lower the signal-to-noise ratio (SNR), the more difficult it is to determine the causal direction for both SOC and GC; the pwLiNGAM still failed in these cases with Gaussian innovations since all the variables are Gaussian. However, when using Laplace innovations to generate data and without Gaussian observational noises (i.e. infinite SNR), the pwLiNGAM and SOC both work well (Fig. 1Ddotted lines). With the increase of the observational noises, SOC and GC outperform the pwLiNGAM and SOC is always better than GC (Fig. 1Dsolid lines).

To summarize, this simulation confirms that the SOC method is able to estimate the proposed model with instantaneous causality and time-dependent variables when the true regressor does not have the same autocorrelation as the noise variable. Compared with GC, SOC performs better, especially in cases where the number of time points is typically quite small and limited. Moreover, pairwise LiNGAM is unable to work when the autocorrelated variables are generated with Gaussian innovations; it only works with non-Gaussian (here Laplace) innovations. Still, SOC outperforms when the data variables are not non-Gaussian enough, for example, because of added Gaussian noise.

### 3.2 Real MEG data

We applied the proposed method to the energies of brain sources obtained from magnetoencephalographic (MEG) signals to analyze their causal relationships. To assess the statistical validity of the results, we first performed a split-half analysis to examine intra-subject consistency, where each subject’s data are split into two epochs and then computed the correlation between the causal measures of two epochs. Second, we also performed inter-subject consistency analysis, where we split the whole set of subjects into two groups and examined the correlation between the causal measures from the two groups. Moreover, we performed brain age prediction using the whole network’s effective connectivity estimated by the two-stage approach (SeeSection 2.3) as the features, to validate the method.

#### 3.2.1 Cam-CAN dataset and preprocessing

We primarily analyzed data from the open-access Cambridge Center for Aging Neuroscience (Cam-CAN) repository (see (Shafto et al., 2014;Taylor et al., 2017) for details of the dataset and acquisition protocols). Specifically, we used the eyes-closed resting-state MEG from 652 healthy subjects (male/female = 322/330, mean age = 54.3 ± 18.6, range 18–88 years), and their structural (T1-weighted MRI) neuroimaging data for source reconstruction. The MRI images were acquired with a 3T Siemens scanner with a 32-channel head coil. The MEG data were recorded using a 306-channel Elekta Neuromag Vectorview (102 magnetometers and 204 planar gradiometers) system at a sampling rate of 1 kHz. For the resting-state scan, subjects were asked to lie still and remain awake with their eyes closed for around 9 min. We discarded the initial 30 s and the final 30 s of data and used the remaining data from each subject for further analysis. Following exclusions (e.g., subjects that did not have both MRI and MEG data, unsatisfactory pre-processing results such as failure to remove cardiac and ocular artifacts, and/or failure to extract the cortical surface for source reconstruction), a final dataset of 610 subjects was retained for further analysis. Regarding the preprocessing, we followed the previous studies (Zhu et al., 2023). Specifically, the MEG data were band-pass filtered to 0.1–40 Hz and resampled at 256 Hz. Cardiac and eye movement artifacts were identified by the FastICA algorithm and removed. After source reconstruction, we parcellated the cortex into 400 Schaefer parcels for each subject and the subjects’ parcel time series were morphed into a standard atlas.

#### 3.2.2 Source separation with nonlinear ICA

To begin with, we separated sources underlying the parcel cortical time series using a recently proposed variant of nonlinear independent component analysis (NICA) (Hyvärinen & Morioka, 2016,^2017^), called independent innovation analysis (IIA) (Morioka et al., 2021). This initial source separation was inspired by a previous two-layer model study (Zhang & Hyvärinen, 2010). Thus, 15 sources were obtained from the well-trained NICA(IIA) model in the previous study (Zhu et al., 2023), where the details could be found. The spatial patterns of the sources can be found in the original paper and in our Supplementary Material (Fig. S1). For the instantaneous causal analysis, we then computed the energies of the sources and resampled the resulting time series at 1 Hz, since the energies change slowly. Specifically, we applied the Hilbert transform to the source time series to obtain the analytic signal and computed the absolute value of analytic signals to derive the amplitude envelope of the energies. For the fast, raw MEG signal, instantaneous causality may not be meaningful; thus, we did not consider it in the current study and prefer to use energies.

#### 3.2.3 Reproducibility analysis on split-half tests

Regarding the two-stage approach to the estimation of the causal networks (Section 2.3), there are two factors that initially affect consistency: consistency of the L1-penalized precision matrix (estimated by cross-validated graphical lasso) and consistency of the causal method (SOC, GC, and pwLiNGAM). The third is the consistency of the two-stage methods combining the two factors. The consistency of causal methods is referred to as the consistency of the raw values of the causal measures in the current study (e.g.,Eq. (6)for the SOC method). We here examine the consistency of all those three metrics.

##### 3.2.3.1 Intra-subject consistency analysis

Each subject’s data (15 source time series) were split into two equal segments (two halves). Then, we estimated the three metrics mentioned above for each epoch separately and computed the correlation between two epochs for each subject.

##### 3.2.3.2 Inter-subject consistency analysis

The whole dataset (610 subjects) was randomly divided into two halves. For each half (305 subjects), the 15 source time series were temporally concatenated across subjects and the concatenated time series were used to estimate the three metrics among the sources’ time series. Then, we calculated the correlation between two groups. This procedure was repeated 100 times.

After the intra- and inter-subject consistency analysis, we further concatenated the all subjects and estimated the causal connectivity networks (causal directions for each pair of connected sources) using the two-stage approach. We considered this group-level causal connectivity networks as a template (population/group level) due to the lack of ground-truth direction. That is, we consider the causal connectivity estimated from the group-level data (temporally concatenated data across all subjects) as a proxy for the ground truth, since such data have a very large sample size. Then, we randomly selected 100 subjects repeatedly from the whole data and computed the causal direction for each pair of connected sources in the template for each subject, which resulted in the subject-specific causal direction. We then calculated the accuracy of the subject-specific causal direction compared with the group-level causal direction (a proxy of ground truth) for each pair of connected sources in the template. Finally, we demonstrated the causal networks showing the accuracy of each pair of connected sources.

#### 3.2.4 Predicting brain age using effectivity connectivity networks

Neuroimaging-driven prediction of brain age was defined as the predicted biological age of a subject using only brain imaging data (here MEG modality). We thus performed brain age prediction using the causality (e.g., effective connectivity) estimated from the 15 underlying sources. To estimate an effective connectivity network of 15 sources, we use a two-stage approach explained above and well known in the literature. First, we estimate which sources are connected using a covariance-based method, such as the L1-penalized inverse covariance. Next, for each pair of connected sources, we estimate the direction using the pairwise approach proposed above. This combination of the information on the existence of connections given by the inverse covariance with the directionality given by our pairwise method gives a method for estimating the whole networks.

We considered the task of predicting the biological brain age using inferred effectivity connectivity networks as features. In the interest of interpretability, we limit ourselves to linear regression models. For model evaluation, we performed standard 10-fold cross-validation based on fixed random seeds, which guaranteed that for any method under consideration, identical data splits were used. The mean absolute error (MAE) and coefficient of determination (R^2^) score were used for scoring prediction performance (Engemann et al., 2022). The R^2^(bigger is better) quantifies the incremental success of a model over a regressor returning the average of the training data as a guess for the outcome. Also, the R^2^metric clearly penalizes systematically wrong predictions by assigning scores smaller than 0. Positive predictive success thus falls into a range of R^2^between 0 and 1 (higher scores are better). MAE has the benefit of expressing prediction errors at the scale of the outcome. This is particularly convenient for scientific interpretation when the outcome has some practical meaning as is the case on age prediction (smaller scores are better) (Engemann et al., 2022).

#### 3.2.5 Statistical analysis of accuracies between methods

To assess the statistical differences between the accuracies or correlation coefficients for different tasks or for different causal methods, we applied the Wilcoxon signed-rank test, FDR correction. Specifically, the individual or single-run accuracies or correlation coefficients were fed into the Wilcoxon rank sum test.

#### 3.2.6 Results

##### 3.2.6.1 Consistency of the split-half analysis

**Fig. 2.**
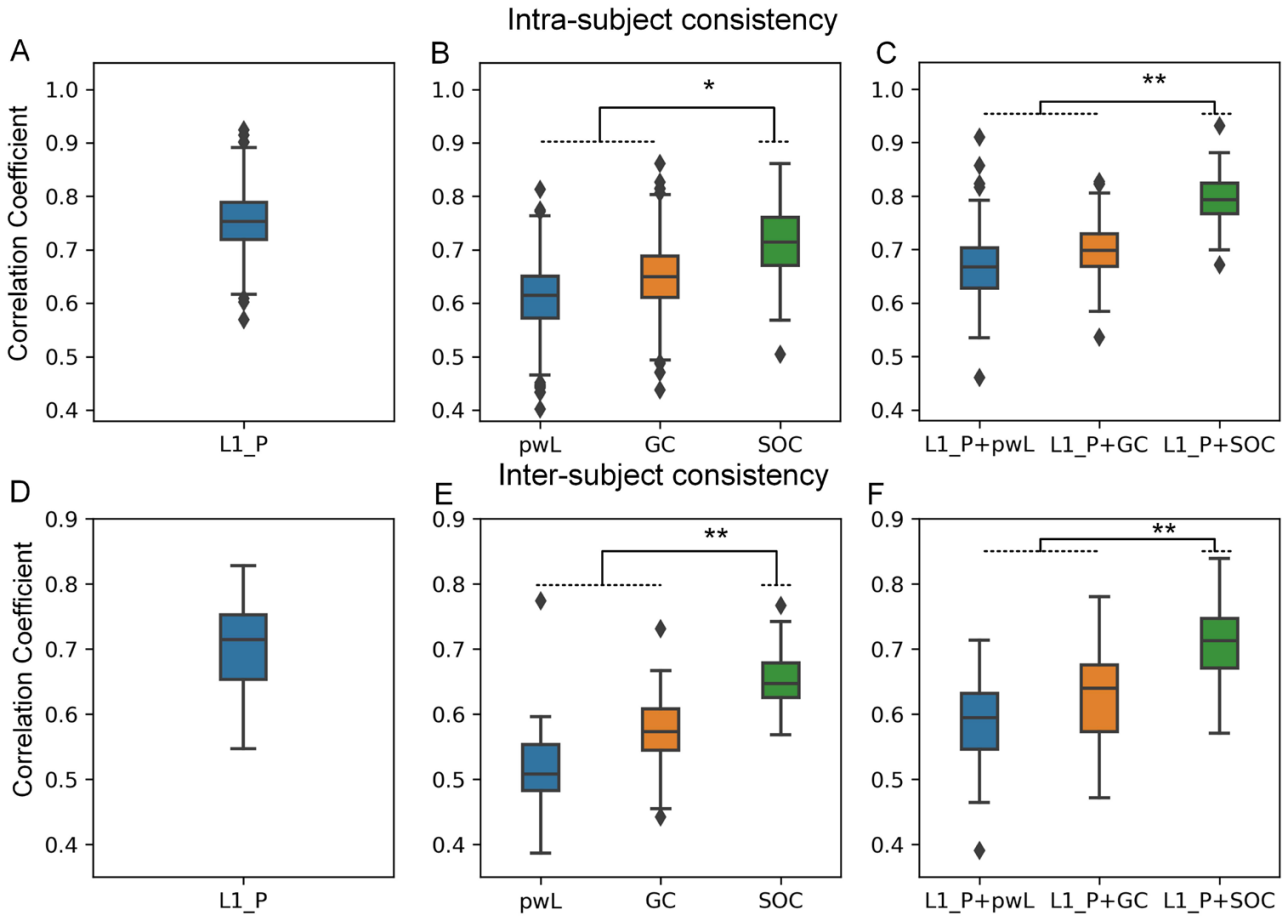
Intra- and inter-subject consistency analysis of split-half tests (the diamond represents outliers, *p < 0.01, **p < 0.001). (A&D) Consistency of L1-penalized precision matrix (cross-validated graphical lasso). (B&E) Consistency of causal methods alone. (C&F) consistency of two-stage approaches (seeSection 2.3).

Figure 2shows the results of intra- and inter-subject consistency analysis of L1- penalized precision matrix, causal methods, and two-stage methods combing the two. In the intra-subject analysis, L1-penalized precision matrix shows a high similarity (quantified by correlation coefficient) between the two epochs (correlation coefficient:0.75±0.05) (Fig. 2A). One can see that all the causal methods demonstrate similarities above 0.5 (Fig. 2B), that is, pwLiNGAM:0.61±0.06, GC:0.65±0.07and SOC:0.71±0.06. When combing the two stages, the similarity between epochs improved for all causal methods (i.e., pwLiNGAM with0.67±0.06, GC with0.70±0.05and SOC0.78±0.04) (Fig. 2C), presumably because the causal methods without L1-penalized precision matrix might measure the consistency in those connections which are only noises. In both cases, the similarities from SOC method were significantly higher than similarities from GC and pwLiNGAM methods (Wilcoxon signed-rank test with FDR correction, seesection 3.2.5).

For the inter-subject analysis, the similarities from all the causal methods (alone or two stage methods) seem to be weaker than intra-subject similarities, perhaps due to inter-subject variability (Fig. 2D-E). L1-penalized precision matrix has similarities between two groups of0.70±0.07(Fig. 2D). For causal methods alone, similarities of pwLiNGAM are with0.52±0.06, GC with0.57±0.06and SOC with0.65±0.05(Fig. 2E). The two-stage methods combing L1-penalized precision matrix also improved the consistencies between two groups, that is, pwLiNGAM:0.59±0.07, GC:0.65±0.08 and SOC:0.71±0.06(Fig. 2F).

**Fig. 3.**
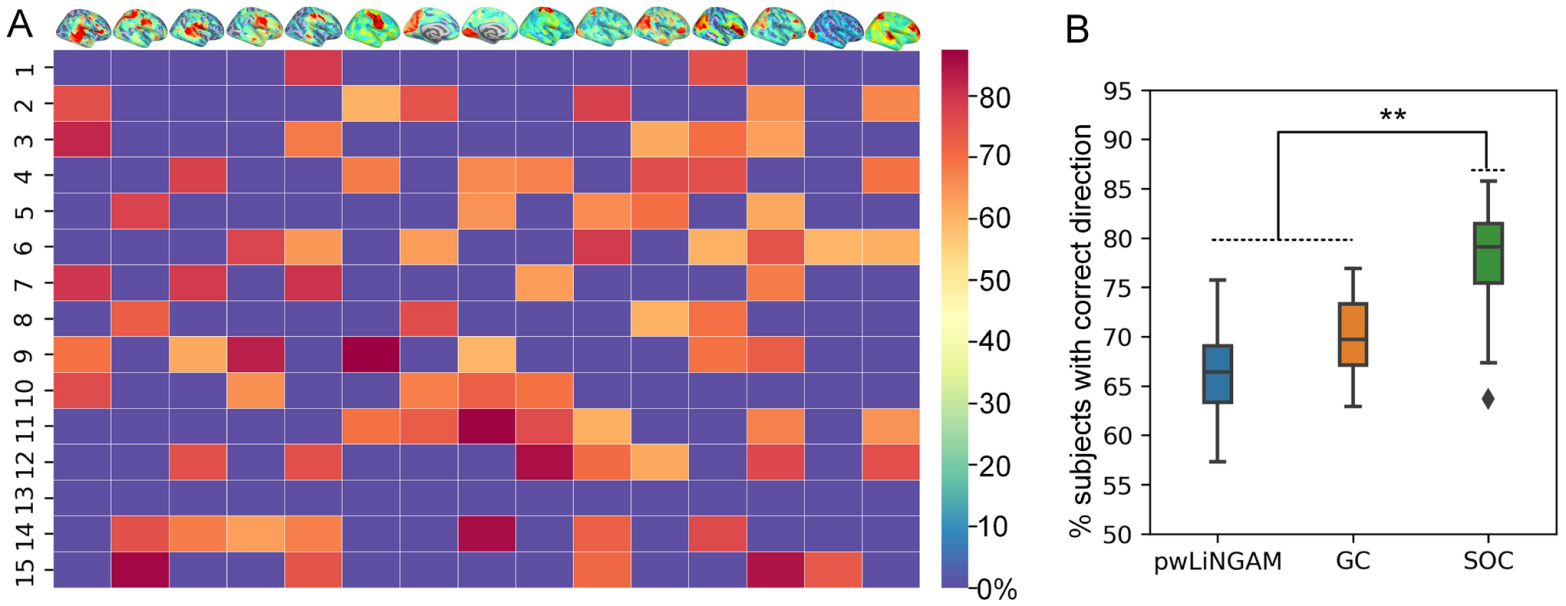
(A) The adjacency matrix indicating the percentage of the number of correct directions from the individuals compared with the templates (a proxy for ground truth). (B) The global percentage of the number of correct directions from individuals which is the same as the template for each pair of connected sources/nodes (the diamond represents outliers, **p < 0.001).

Figure 3Ashows the adjacency matrix indicating the accuracies of correct direction estimated on the subject level (percentage of subjects equal to the group-level template) for each pair of connected sources in the group-level template (which is considered a proxy for ground truth). One can see that basically causal direction estimated from most subjects was consistent with the template for each pair of connected sources (around 75% accuracies, chance level 50%).Figure 3Bshows the comparison with other methods by randomly selecting 100 subjects from the whole data and computing the accuracies related to the template. We can see the accuracies (0.79±0.05) from the proposed SOC were significantly higher than other methods, pwLiNGAM with0.66±0.04and GC with0.7±0.03.

##### 3.2.6.2 Group-level effective connectivity networks

**Fig. 4.**
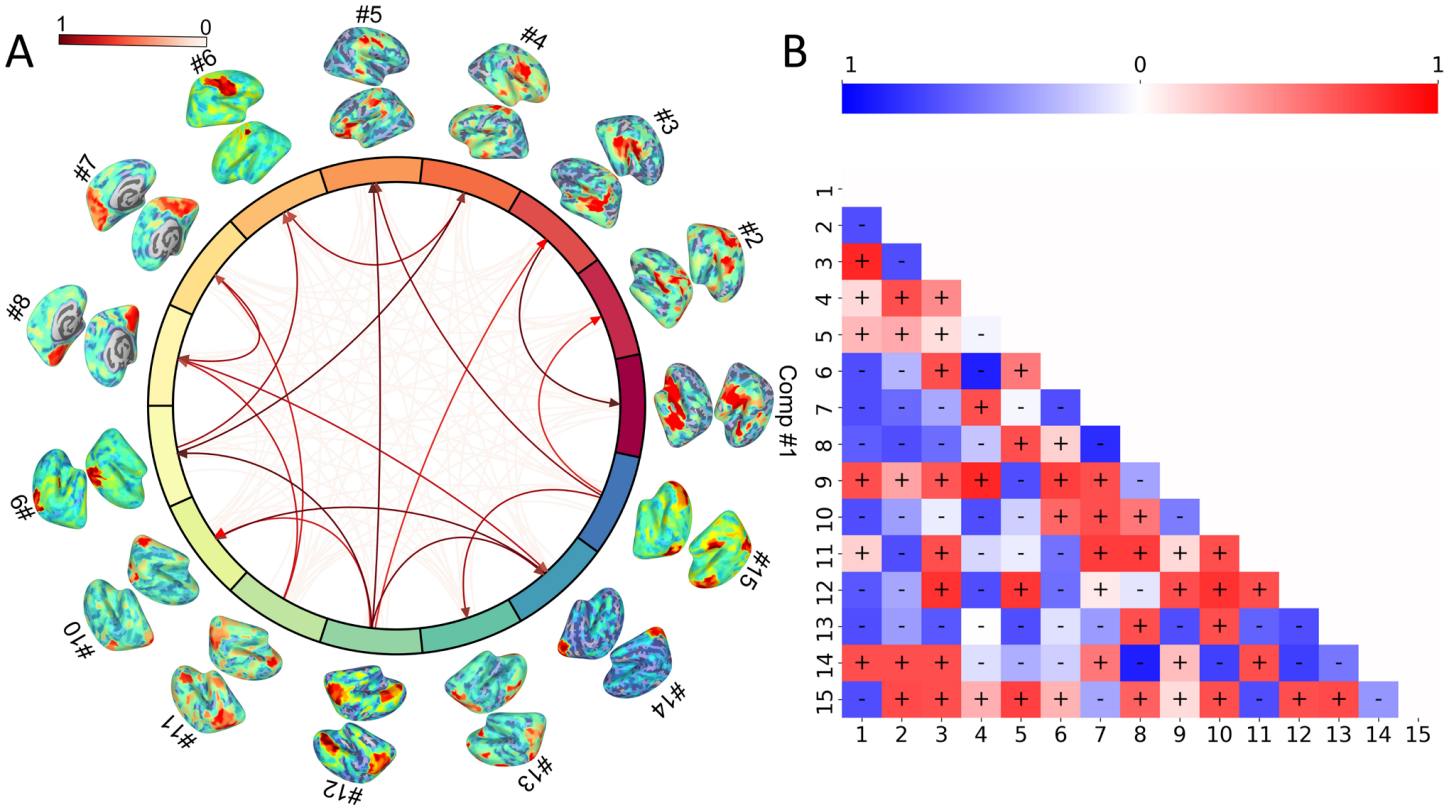
Directional connectivity networks derived from the concatenated MEG sources across the whole subjects. (A) The top 5% (just for better visualization) connections are visualized, and the arrows represent causal direction. The color of the line represents the absolute causal measures inEquation (6). (B) The heatmap shows the causal directionality, where the “-” sign in the blue box represents the flow into the current node and the “+” sign in the red box represents the flow out. The colorbar represents the amplitude of causal measures.

Figure 4shows the resulting effective (or causal) connectivity networks of causal analysis with instantaneous effects between the energies of the MEG sources (two-stage analysis with proposed SOC methods on a whole set of subjects), with the influences significant at 5% level. One can see that the connections tend to be strong between sources, which are close to each other (Fig. 4A-B). For example, the visual patterns of sources such as #7, #8, #11, and #14 have strong interconnectivity, which might be related to visual processing and possibly spatial attention. Auditory sources such as #1 and #3 are also strongly interconnected. Some sources related to sub-regions of sensorimotor and dorsal attention networks, including #4, #6, and #9, have strong interconnectivity. Some sources involved in the regions of default mode networks and control networks seem to have an impact on the visual, motor, and auditory sources, since those networks are associated with the voluntary, top-down deployment of attention (Fig. 4B). For example, source #15 has influences on sources #2, #5, and #13; source #12, engaged in sub-regions of control networks, has influences on sources #3, #9, #10, and #14.

##### 3.2.6.3 Brain-age prediction

**Fig. 5.**
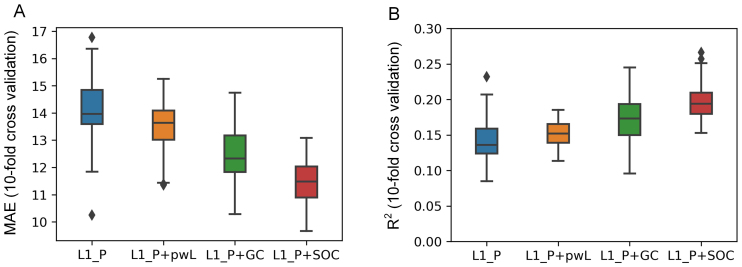
Brain age prediction using the effective connectivity features (Precision: L1-penalized precision matrix; pwLiNGAM: pairwise LiNGAM; Granger: standard Granger Causality; and SOC: the proposed second-order causal discovery method). Generalization performance was assessed by 10-fold cross-validation. (A) The MAE years (smaller is better) and (B) the R2 score (bigger is better) were compared for the different methods (the diamond represents outliers).

We compared the performance of all causal discovery methods using a 10-fold cross-validation approach and repeated the cross-validation framework 10 times. A summary of the performance of the different brain age prediction methods is presented inFigure 5. One can see that prediction using effective connectivity features from SOC method yielded best performance with a mean absolute error (MAE) of11.3±1.8 years, which was significantly lower (Wilcoxon signed-rank test, seeSection 3.2.5) than GC (MAE of12.5±2.1 years) and pwLiNGAM (MAE of13.6±2.7 years) methods (Fig. 5A) and only using L1 penalty sparse precision matrix (cross-validation graphical lasso estimator) (MAE of14.0±2.8 years) as well.Figure 5Bdemonstrates that all methods have positive R^2^scores, which implicates correct predictive success. One can see that the proposed SOC method has higher R^2^scores (0.2±0.05) than others, GC with R^2^of0.17±0.06, pwLiNGAM with R^2^of0.15±0.04, and L1 penalty precision matrix with R^2^of0.14±0.07.

## 4 Discussion

In this study, we proposed an instantaneous causality analysis for energies or envelopes of MEG sources or fMRI signals using the second-order blind source separation method. It allowed us to derive a measure for effective or causal connectivity between separated brain regions, which was proven to be superior to traditional functional connectivity analysis (e.g., standard Granger Causality and LiNGAM methods) in some cases. The proposed method only exploits the temporal dependence (i.e., autocorrelation), not the non-Gaussianity for example, and is suitable for instantaneous causal analysis of neuroimaging data such as fMRI and energies of E/MEG. Importantly, many popular neuroimaging preprocessing methods produce near-Gaussian time series, while autocorrelations are ubiquitous. In such cases, some popular causal discovery approaches that require the data to be non-Gaussian, such as LiNGAM-based methods, would fail. Also, Granger-causality methods fall short in handling instantaneous (contemporaneous) effects between sources since they are based on the observation that cause occurs prior to its effect. Experiments with both simulated data and real MEG data validated our method and demonstrated that such an approach is able to find the causal direction from the autocorrelated time series better than non-Gaussianity-based methods and the time-lag-based Granger method. It should be noted that, despite the Gaussian assumption, the proposed method can be applied to non-Gaussian signals (seeFig. 1DandFig. S6in the Supplementary Material). It is well known in the context of generative model and causal inference that the theoretical assumption of Gaussianity does not really mean that the data must be Gaussian. The point is that a Gaussian distribution is completely characterized by the covariances (and the mean), and “assuming” Gaussianity actually means that we are restricting the analysis to covariances (and the mean) only. Any non-Gaussianity would only affect the higher-order moments, not the second-order moments or covariance. Thus, non-Gaussianity would be rather irrelevant in fitting a Gaussian model. It would slightly decrease the efficiency of the model estimation but would not make it biased. We also wish to emphasize that our study is not primarily focused on introducing a completely new theory. Rather, the core contribution of our work lies in applying the method, based on likelihood ratios related to mutual information, specifically to instantaneous causal inference for the energies of MEG data. Although information-theoretic approaches have been applied in causal inference research more broadly, there has been limited application of these methods in the context of instantaneous causal analysis for MEG data.

In the simulation experiments, no matter what conditions, we observed the performance of the proposed SOC method for finding the correct pairwise direction is clearly superior to the standard Granger Causality method, especially when the number of data points is small (Fig. 1A-D), since Granger causality occurs when patterns in time series data occur in another time series after some time lags. Meanwhile, in those cases where Gaussian innovations were adopted to generate variables in the AR process, the pwLiNGAM method failed to determine the direction since the distribution of the data was Gaussian or not non-Gaussianity enough. However, when using the Laplace innovation to generate the variables without adding observational Gaussian noise, pwLiNGAM performs best since Laplace innovations in the AR process result in non-Gaussian data. However, when observational Gaussian noise is added, and as the intensity of the Gaussian noise increases (SNR decreases), thus weakening the non-Gaussianity of the data, the performance of pwLiNGAM becomes worse until the SOC and GC outperform it (Fig. 1D). These results show that the SOC method has the potential to be an effective tool for causal analysis when the data sample sizes are limited, which is very common in practice. For example, it is not easy to obtain enough neuroimaging data in some cognitive neuroscience paradigms, such as fMRI and MEG. Moreover, our SOC method makes the causal direction identifiable if and only if the regressor variable does not have the same spectrum as the noise in the model, which was shown in the simulation (Fig. 1B). This is consistent with the second-order statistic based blind source separation method. If the variable and noise in the model have the same autocorrelation spectra, any estimation method will fail for our causal model, which is one important limitation. It should be noted that we adopted the AR model to generate the data here, rather than other biophysical models, such as the neural mass model (David & Friston, 2003), because our primary consideration was for a model capable of producing synthetic data with controlled temporal dependencies rather than biological realism. While biophysical models, that is, the neural mass model, would, indeed, generate more biologically realistic signals, they would introduce additional complexity and numerous biophysical parameters that are not directly relevant to the method.

When applying to real MEG data, due to the lack of ground truth of the causality of MEG sources, it is very difficult to quantify the improvement of the proposed method. We here validated the performance of the method by examining the intra-subject consistency and inter-subject consistency between the two halves of the data. For the intra-subject analysis (Fig. 2A-C), GC and pwLiNGAM performed not well since the sample sizes were small in this case, where the SOC performed quite well. When combining the L1-penalized precision matrix with causal measures into two-stage approaches, the consistency across two epochs improved since the L1-penalized precision matrix removed those connections measured by the causal method alone which might be noise. We here adopted the L1-penalized precision matrix since the consistency of the L1-penalized precision matrix is higher than the L2-penalized method (seeFig. S4). Regarding the inter-subject analysis (Fig. 2D-F), the consistencies of all the methods, causal methods alone or two-stage methods, seem to be weak compared to the intra-subject analysis, although still acceptable. This might be due to the inter-subject variability on one hand and the increase of the sample sizes (concatenated across subjects) for the estimation on the other hand. These results demonstrated that all the methods basically had reasonable intra-subject and inter-subject consistencies (correlations between two halves are higher than 0.5), while the SOC method performed significantly better than the others. For the further consistency analysis at a population level, the results also demonstrated reasonable intersubject consistencies (the accuracies compared with group-level template (a proxy for ground truth) are higher than the chance level of 50% for each pair of connected sources (Fig. 3)).

We also show how pairwise measures can be used to estimate the whole effective connectivity (causal connectomes) networks for neuroimaging data in a two-stage method. Based on such a method, we demonstrated the effective connectivity networks for the nonlinear MEG sources (Fig. 4), which allowed us to recover the information flow among the whole brain from the data and beyond what is possible with traditional functional connectivity methods (Rawls et al., 2023). We observed that similar spatial patterns of the sources tend to be causally influenced, and the sources involved sub-regions of higher cognitive networks, such as control, attentional, and default mode networks had influence on other sources related to sensorimotor, auditory, and visual networks. These sources had distinctly different causal connectivity profiles. For example, sources associated with attentional networks shared incoming connections with sources involved in sensory regions and outgoing connections with sub-regions of higher cognitive networks, while sources involved in executive networks primarily connected to other sub-regions of higher cognitive networks, which is consistent with previous study (Rawls et al., 2022).Figure 3also demonstrated the consistency of the intersession for such effective connectivity networks estimated by the proposed method, in which the correlation coefficients between them were clearly quite large.

We also validate the method via a brain age prediction task, since it is widely accepted that aging has pronounced effects on the functional architecture of the human brain. While we noted that brain age prediction errors presented in this work are not competitive with alternative methods which are based on alternative imaging modalities, such as structural imaging data and fMRI (Cole & Franke, 2017;Monti et al., 2020;Mouches et al., 2022;Yu et al., 2024), the results from MEG data in the current study validated that the proposed SOC method showed significantly better performance than other methods (Fig. 5). The MAE scores observed across methods of 11 to 13 years have been larger consistently reported in the literature. For example, Engemann et al reported a benchmark of brain age prediction from MEG resting-state data, where a couple of methods, such as deep learning based, handcrafted features based, and covariance-based filterbank approaches, were used and shown the MAE scores of the Cam-CAN dataset with 8 to 10 years (Engemann et al., 2022). However, MAE scores in the current study are lower than the chance level of 16 years quantified by the dummy model (Engemann et al., 2022), and the R^2^scores are positive, implicating the rationality of the current results. These results also indicate that the effective connectivity networks estimated by the proposed method from MEG might be considered as neuroimaging-derived features to predict the biological brain age. While the brain-age prediction results (evaluated by MAE and R scores) using the proposed SOC method showed significantly better performance than baseline methods, it looks not competitive with deep learning methods. It should be noted that here our method for the brain age prediction task is quite simple and likely to provide complementary information that could be combined with those used by (Engemann et al., 2022). Several factors can influence the performance of brain age prediction in terms of the current framework. Effective connectivity used as features capture directed causal interactions between brain regions, and their effectiveness in age prediction relies heavily on how well these causal interactions reflect underlying age-related changes in brain function. In the current study, we use the intra-subject and inter-subject consistency analysis to evaluate the sensitivity or stability of causal discovery methods, and the higher the consistency, the more stable or reliable the causal directionality estimated is. Rather encouragingly, we see that the higher consistency of the method (SOC, pwLiNGAM, or GC) correlates with better in brain-age prediction (compareFigs. 2&5).

Here, we examined the causality among the nonlinear ICA sources instead of, for example, parcellated time series, since the nonlinear ICA procedure could be considered as combined source separation and dimension reduction. In future studies, we could integrate such two steps, nonlinear source separation and causal discovery, into one framework, which is called causal representation learning in the literature (Schölkopf et al., 2021). We have actually further performed the causal analysis on the energies of parcel time series, with the proposed method, instead of the underlying nonlinear ICA sources. The results of intra- and inter-subject consistency analysis are shown in the Supplementary Material (Fig. S2), showing that the consistencies are much better with nonlinear ICA sources (Fig. 2) than with parcellated data. This might be due to lots of factors, such as, how the parcellation is chosen (e.g., fine- or coarse-scale parcellation), how it is post-processed, and especially here the large number of parcels (400 Schaefer-parcels) might result in spurious connections. Thus, regarding the larger number of parcels, we re-parcellated the brain with a coarse-scale solution that organized the cortex into 7 networks in each hemisphere (Yeo2011_7Networks Atlas), resulting in 14 functional regions in the whole brain (Yeo et al., 2011), close to the number of nonlinear component sources. Then, we performed the causal analysis on the energies of the 14 parcel time series. Finally, we also obtained a quite large intra- and inter-subject consistency in the half-split tests (Fig. S3), which is consistent with the results of causal analysis on 15 nonlinear sources (Fig. 2). This seems to imply the effectiveness of the proposed causal analysis on parcel data, if the parcels are well chosen.

Additionally, we chose not to include the Dynamic Causal Model (DCM) in our comparison because DCM (Friston et al., 2003,^2019^) is quite different in its approach: it is a model- and hypothesis-based method, whereas our proposed method is kind of data-driven. Specifically, DCM relies on predefined models of interactions and prior assumptions about the underlying network topologies, which may not be appropriate or feasible for certain types of data where structural topologies are difficult to validate. In other words, it is unable to effectively search across the full range of possible network topologies. The second reason why DCM is not included here is that we are interested in analyzing resting rather than task MEG data. Although some preliminary work on stochastic DCMs suggests it could potentially be applied in this context, the standard DCM methods require that the “input” timings be specified in advance—something that is clearly not known for resting data (Smith et al., 2011). In fact, our simulation results suggest that DCM does not perform well in the current simulated setting (seeFig. S7in Supplementary Material).

Regarding the backward connections, it is rarely the case that two brain regions are connected in one direction only; there will generally be connections in both directions, including those with predominantly feedforward or backward connections. However, even if a connection is bidirectional, the connection is unlikely to have the same strength in both directions, and causal discovery like the proposed SOC method is likely to point out the stronger direction.

To conclude, we presented a model and corresponding estimation approach for instantaneous causal discovery in time-dependent series for Gaussian autocorrelated variables based on second-order blind source separation methods, which provides an alternative to non-Gaussianity-based methods as in LiNGAM. The autocorrelations make the model identifiable without explicit prior assumptions on the direction or existence of causal effects. The proposed measures seem to be particularly useful in the case where the number of data points is small compared to the dimension of the data, or the data are noisy. In such a case, the statistical performance of our methods is clearly superior to the Granger-causality methods and the LiNGAM methods. Since our method uses only correlations and not non-Gaussianity, it could be particularly useful for neuroimaging data such as fMRI, or energies of E/MEG data, which follow practically contemporaneous (i.e., instantaneous) causal relationships, due to their low temporal resolution. Hopefully, the proposed methods will allow for finding causal connectivity patterns that are common to all, or most, brains.

## Supplementary Material

Supplementary Material

## Data Availability

The data used in the manuscript are from the Cambridge Centre for Ageing and Neuroscience repository (Cam-CAN;https://www.cam-can.org/). The implement of the SOC code will be found on the first author’s website (https://github.com/yongjiezhu/soca).
