# Supplementary material for: Second-order instantaneous causal analysis of spontaneous MEG
Source: Imaging Neurosci (Camb). 2025 Apr 25;3:imag_a_00553. doi: 10.1162/imag_a_00553 (PMC12320013; doi:10.1162/imag_a_00553)
Supplement: Supplementary Material [file imag_a_00553-supp.pdf]

Second-order instantaneous causal analysis of spontaneous MEG

Yongjie Zhu [a, b*], Lauri Parkkonen [b] and Aapo Hyvärinen [a]

[a] *Department of Computer Science, University of Helsinki, 00560 Helsinki, Finland*

[b] *Department of Neuroscience and Biomedical Engineering, Aalto University, 00076 Espoo, Finland*

*Corresponding author: yongjie.zhu@helsinki.fi

Supplementary Material

1. 15 sources estimated by nonlinear ICA

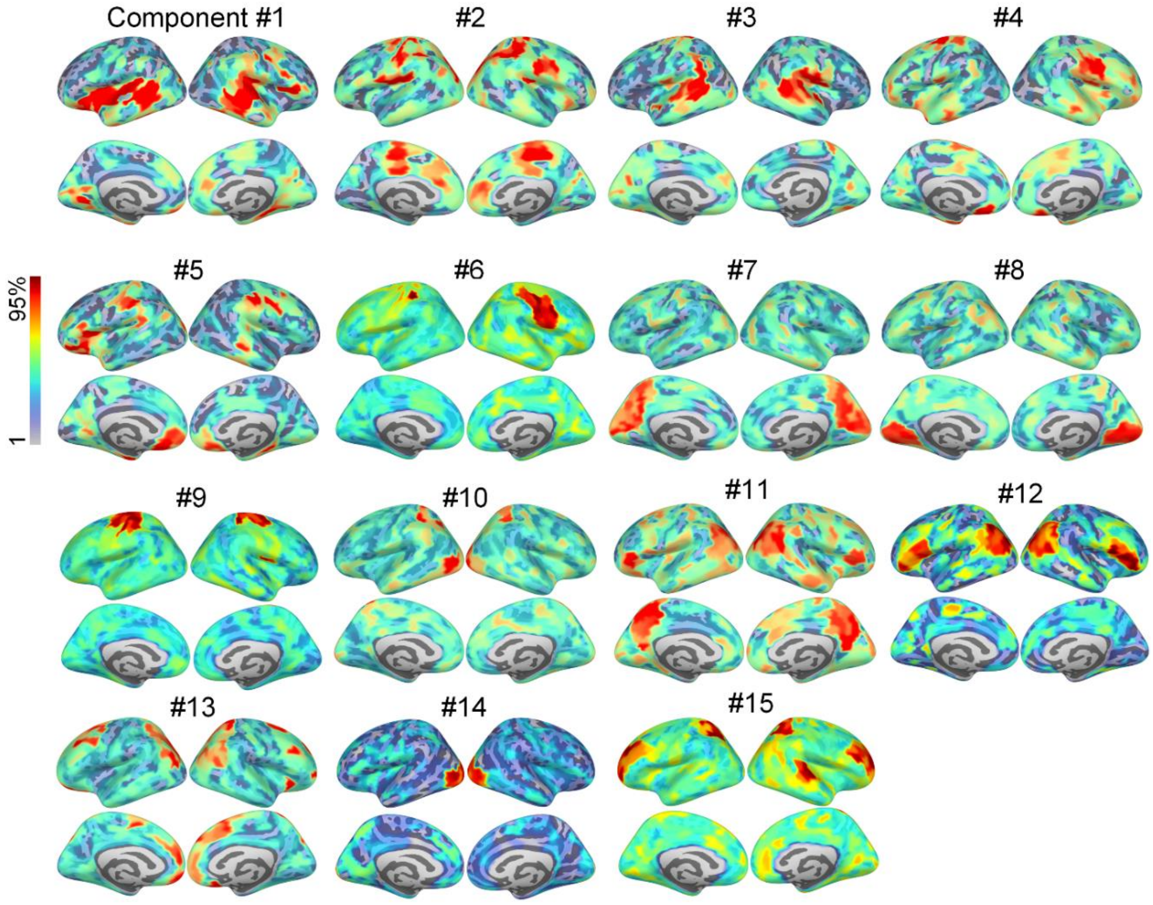

Figure S1. The spatial patterns of all the 15 components estimated by NICA(IIA) from resting-state MEG in the Cam-CAN (Adopted from (Zhu, Parviainen, Heinilä, Parkkonen, & Hyvärinen, 2023)).

2. Causal analysis on energies of MEG signals with 400 parcels

We performed the same intra-subject and inter-subject consistency analysis on the energies of parcellated time series (400 Schaefer-parcels) before nonlinear ICA (as Section 3.2.3). And the results were shown in Figure S2 as follow. The consistencies of all the SOC methods were significantly higher than GC and pwLiNAGM methods. The consistencies are much better with nonlinear ICA sources (Figure 2) than with parcellated data. This might be due to lots of factors, such as, how the parcellation is chosen (e.g., fine- or coarse-scale parcellation), how it is post-processed, and especially here the large number of parcels (400 Schaefer-parcels) might result in spurious connections.

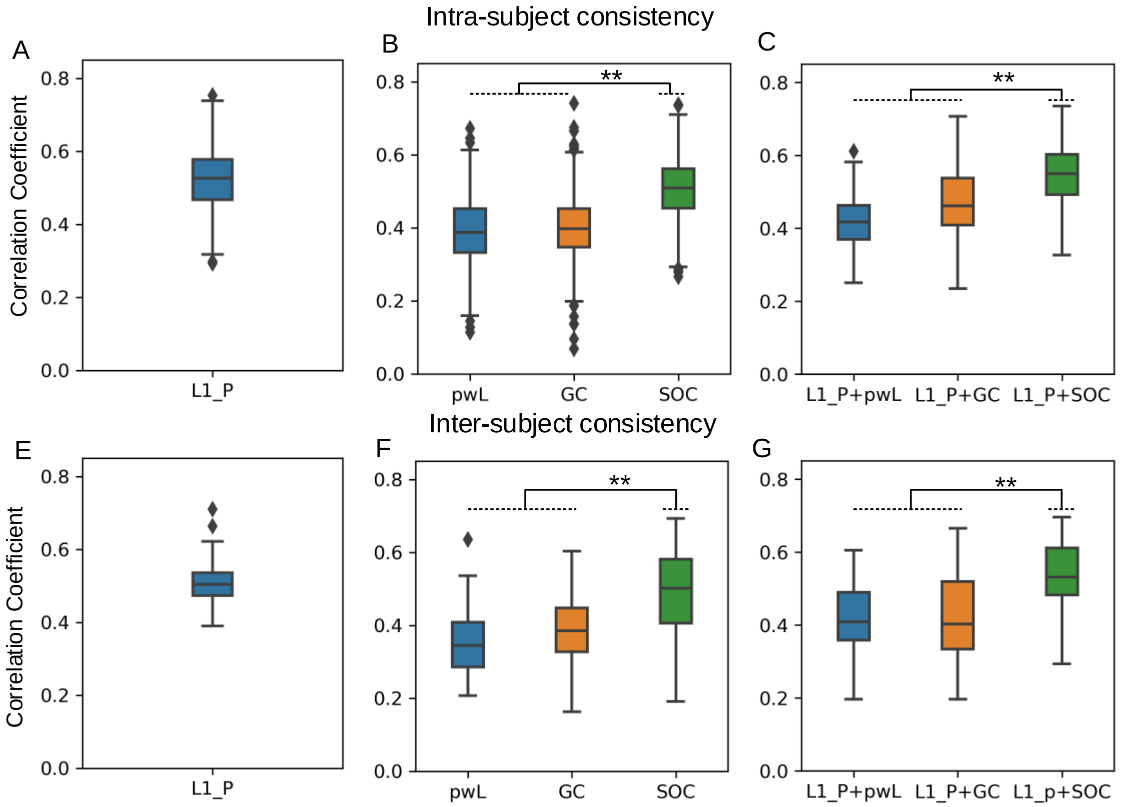

Figure S2: Intra- and inter-subject consistency analysis of split-half tests (parcellated time series from 400 Schaefer-parcels). A&E: Consistency of L1-penalted precision matrix (cross-validated graphical lasso). B&F: Consistency of causal methods alone. C&G: consistency of two-stage approaches ($p < 0.001$ **).

3. Causal analysis on energies of MEG signals with 14 parcels

We re-parcellated the brain with a coarse-scale solution that organized the cortex into 7 networks in each hemisphere (Yeo2011_7Networks Atlas), resulting in 14 functional regions in the whole brain (Yeo et al., 2011), close to the number of nonlinear component sources. Then we performed the causal analysis on the energies of the 14 parcel time series. Finally, we also obtained a quite large intra- and inter-subject consistency in the half-split tests (Figure S3), which is consistent with the results of causal analysis on nonlinear sources.

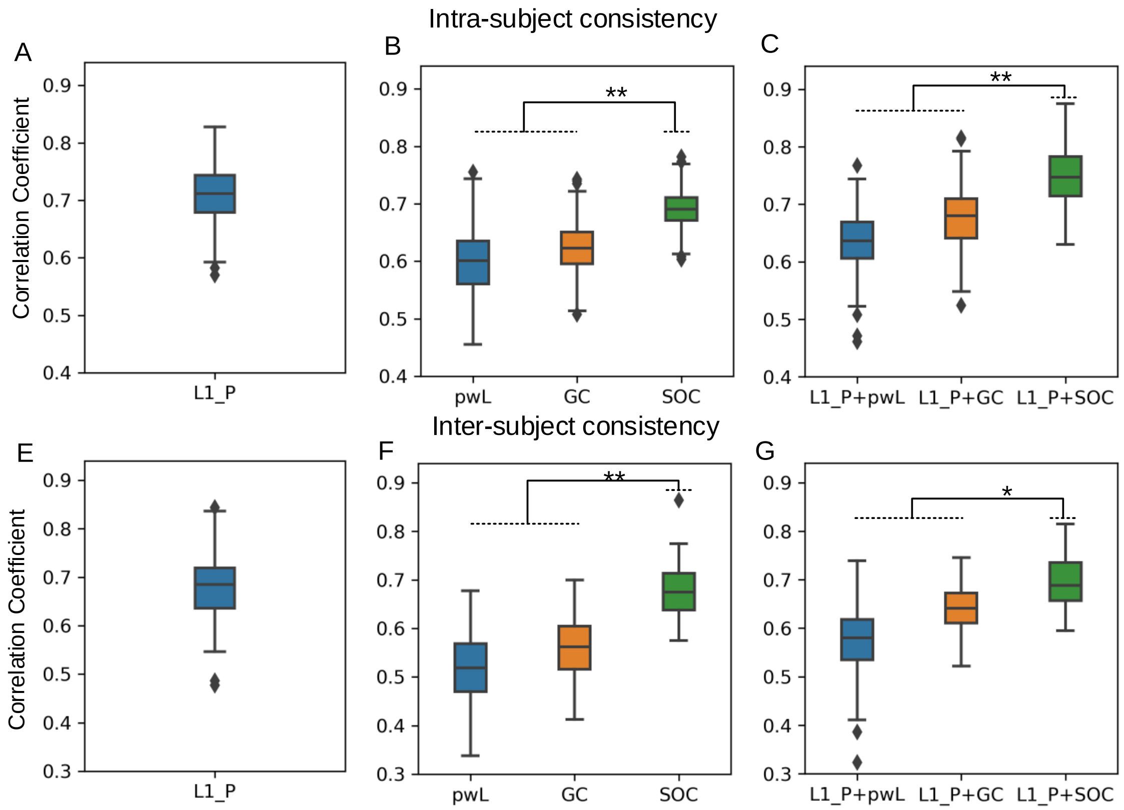

Figure S3: Intra- and inter-subject consistency analysis of split-half tests (parcellated time series from a coarse-scale parcellation with 14 parcels). A&E: Consistency of L1-penalted precision matrix (cross-validated graphical lasso). B&F: Consistency of causal methods alone. C&G: consistency of two-stage approaches ($p < 0.01$ *, $p < 0.001$ **).

4. Consistency analysis for L1- and L2- penalized precision matrix

We also explored the use of an L2 penalty to compute the precision matrix and analyzed the intra-subject and inter-subject consistency. The results showed that the consistency obtained with the L2 penalty approach was lower than that achieved with the L1 penalty. This may be because, although the L2 penalty is loosely regularised it introduces numerous spurious connections, leading to suboptimal results.

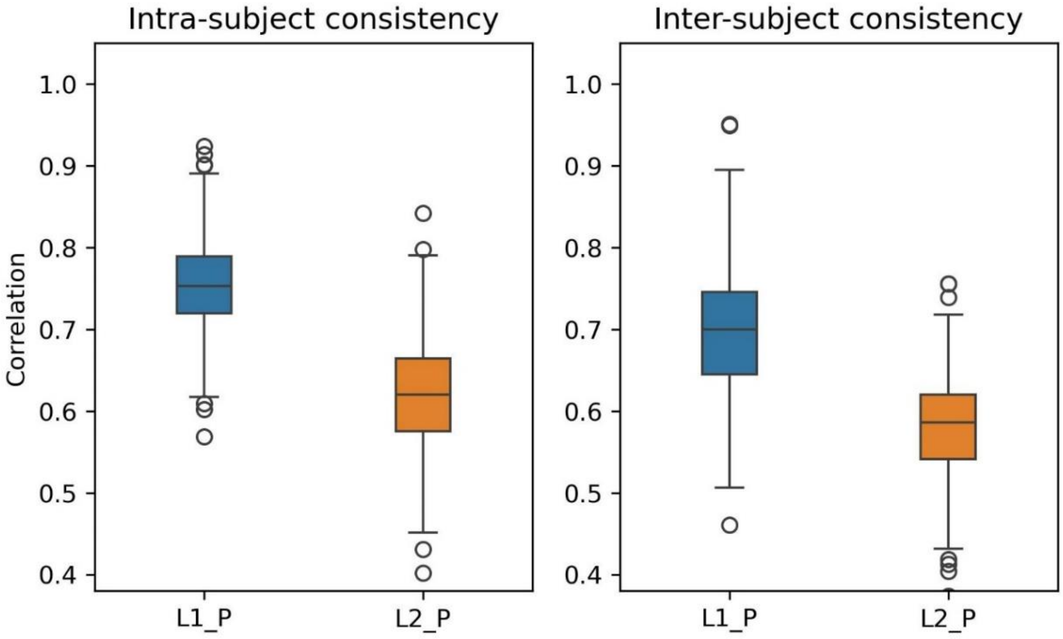

Figure S4. Consistency analysis for L1- and L2- penalized precision matrix.

5. Results from simulated data with AR(3) model

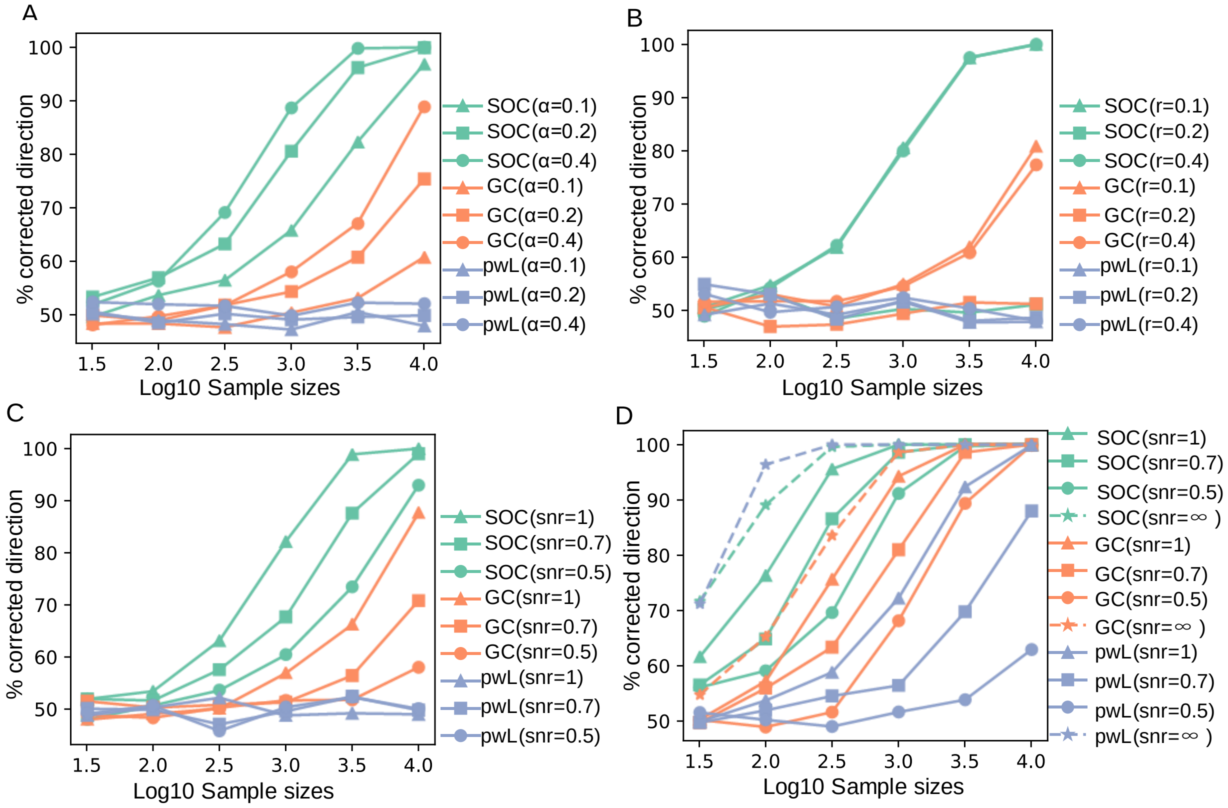

Figure S5. Results from simulated data with AR(3) model, where we set the coefficients r1=r2=r3 for simplicity.

6. Histogram of MEG from one subject

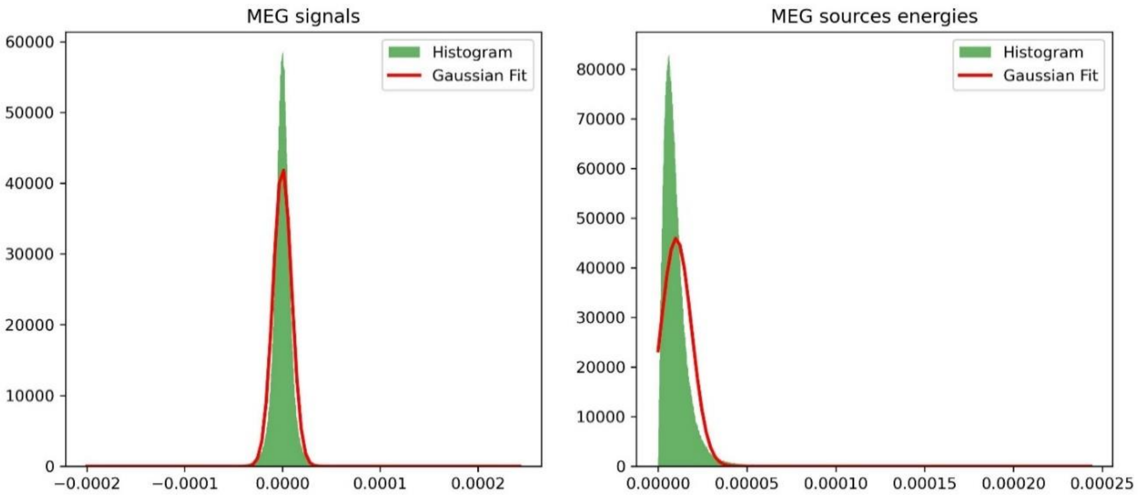

Figure S6. Histogram of MEG from one subject. Gaussian fit refers to a fitted Gaussian distribution that is overlaid on the histogram of data (same mean and standard deviation).

7. The simulation results of DCM

We conducted additional simulations using the DCM framework implemented in the SPM MATLAB package. Specifically, we focused on a resting-state scenario by excluding modulatory and external inputs in the parameter setting. We defined a simple X->Y causal structure to match our simulated ground truth used in other methods. Then we performed DCM estimation and computed the accuracy of the inferred causal direction. Due to the high computational complexity of DCM, we limited our comparison to a single representative condition from each case in Figure 4(ABCD). The results indicate that DCM seems to fail to reliably infer the correct causal direction in our simulated setting. This might be due to the fact that DCM is designed primarily for task-based studies with known experimental inputs, whereas our method is optimized for data-driven analysis of resting-state signals.

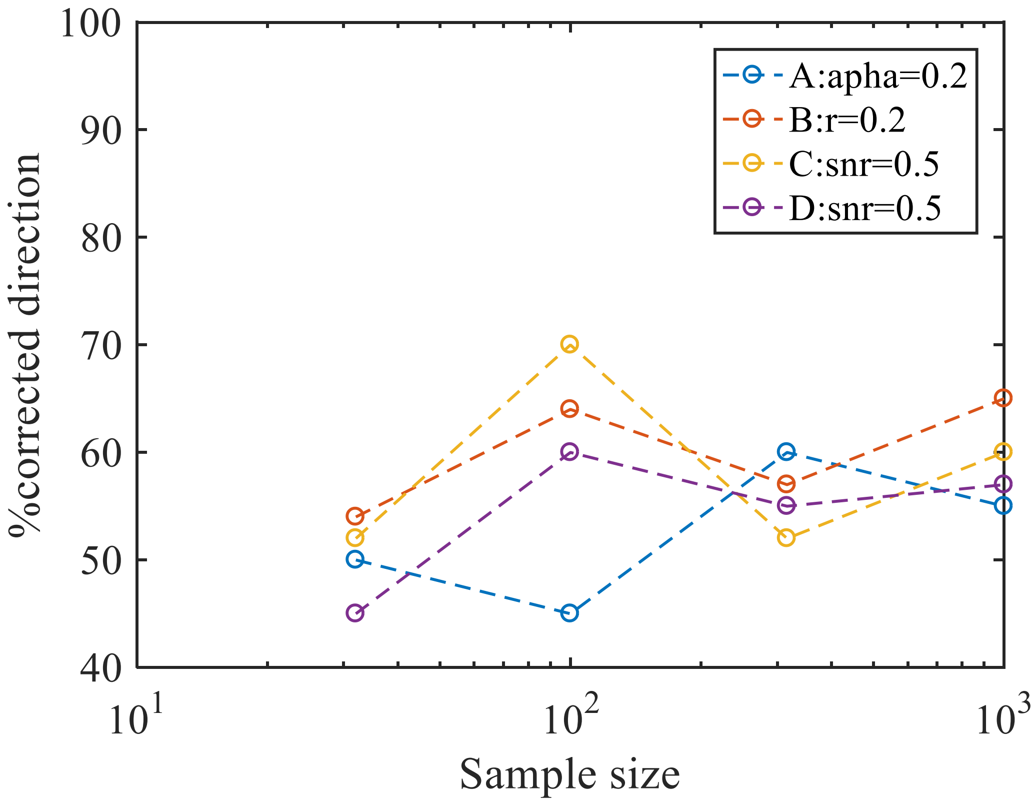

Figure S7. The simulation results of DCM. A. The regression coefficients alpha =0.2. B. The autocorrelation r=0.2. C: Gaussian innovations in AR model and with Gaussian observational noises. D: Laplace innovations in AR model and with Gaussian observational noises.

References

Yeo, B. T., Krienen, F. M., Sepulcre, J., Sabuncu, M. R., Lashkari, D., Hollinshead, M., . . . Polimeni, J. R. (2011). The organization of the human cerebral cortex estimated by intrinsic functional connectivity. *Journal of neurophysiology, 106*(3).

Zhu, Y., Parviainen, T., Heinilä, E., Parkkonen, L., & Hyvärinen, A. (2023). Unsupervised representation learning of spontaneous MEG data with nonlinear ICA. *Neuroimage, 274*, 120142.
